# The Automatization of the Gait Analysis by the Vicon Video System: A Pilot Study

**DOI:** 10.3390/s22197178

**Published:** 2022-09-21

**Authors:** Victoriya Smirnova, Regina Khamatnurova, Nikita Kharin, Elena Yaikova, Tatiana Baltina, Oskar Sachenkov

**Affiliations:** 1 Institute of Computational Mathematics and Information Technologies, Kazan Federal University, 420008 Kazan, Russia; 2 N.I. Lobachevsky Institute of Mathematics and Mechanics, Kazan Federal University, 420008 Kazan, Russia; 3 Interdisciplinary Neuroscience Faculty, Goethe-Universität Frankfurt am Main, 60323 Frankfurt am Main, Germany; 4 Institute of Engineering, Kazan Federal University, 420008 Kazan, Russia; 5 Neurosurgical Department, Central City Clinical Hospital, 432017 Ulyanovsk, Russia; 6 Institute of Fundamental Medicine and Biology, Kazan Federal University, 420008 Kazan, Russia; 7 Department Machines Science and Engineering Graphics, Tupolev Kazan National Research Technical University, 420111 Kazan, Russia

**Keywords:** gait, step phases, clustering high-dimensional data, Vicon, machine learning

## Abstract

The quality of modern measuring instruments has a strong influence on the speed of diagnosing diseases of the human musculoskeletal system. The research is focused on automatization of the method of gait analysis. The study involved six healthy subjects. The subjects walk straight. Each subject made several gait types: casual walking and imitation of a non-standard gait, including shuffling, lameness, clubfoot, walking from the heel, rolling from heel to toe, walking with hands in pockets, and catwalk. Each type of gait was recorded three times. For video fixation, the Vicon Nexus system was used. A total of 27 reflective markers were placed on the special anatomical regions. The goniometry methods were used. The walk data were divided by steps and by step phases. Kinematic parameters for estimation were formulated and calculated. An approach for data clusterization is presented. For this purpose, angle data were interpolated and the interpolation coefficients were used for clustering the data. The data were processed and four cluster groups were found. Typical angulograms for cluster groups were presented. For each group, average angles were calculated. A statistically significant difference was found between received cluster groups.

## 1. Introduction

Today, there are many reasons why a person’s motor functions are impaired, in particular, a normal gait worsens. Such diseases include blood stroke after which one side of the body is partially or completely paralyzed. Disabled persons with spinal cord injury in various regions, patients with scoliosis, osteochondrosis, hemiparesis (unilateral paresis) of cerebral genesis [1,2], etc. have similar problems. Infantile cerebral palsy also influences movement disorders. Gait features depend on the physiological characteristics of a person, age, and mental state [3,4]. Gait analysis is an important clinical tool for planning clinical treatments. It is being actively introduced into the sport industry to predict injury.

There are various studies in this field. Locomotion research is important for a patient’s diagnosis. D. Kaplun et al. [5] describe an automatic orientation algorithm based on the alignment of the virtual anatomical axis of the lower leg of a 3D model with the vertical axis of rectangular coordinates in a three-dimensional space. The accuracy of the orientation of the stump greatly influences the patient’s locomotion. A digital twin was obtained with the 3D. The spatial orientation of the model was determined by the angles between the projections of the anatomical axis on the plane of the Cartesian coordinate system. To rotate a digital 3D model, a set of transformations was used following the approach of rotation matrices. In 3D space, rotation around the Z axis is described by a matrix transformation. The development shows that the use of a 3D model simplifies and speeds up the process of solving physiological problems.

M. Baltin et al. [6] studied changes in the range of motion of the hind limbs of rats induced by epidural stimulation in the acute phase of SCI and recovery of movements was assessed. A kinematic analysis of complete stepping cycles was performed for animals of each group. Vicon Nexus 2.5 software was used to manually complete the 3D motion model and remove artifacts from the recording. In each group of test rats, the data of the average values of 30 steps in the angles in the phases of one step were researched. For each group of test rats, the average angles (beyond 30 steps) for each step phase were researched. The data were obtained in the form of angulograms. This approach is convenient for visualizing the change of the values of the angles in the phases of the step, as well as for further statistical analysis.

Research by Hiroaki Hobara et al. [7] investigates the movement of a person with prostheses upstairs. The study was carried out using an eight-camera motion analysis system (VICON 512, Oxford Metrics, Oxford, UK). Kinematic parameters such as the angle at the hip and knee (and their swing average flexion rate) were calculated. During the swing, the average flexion rate of the hip and knee was found. Changes in angles in the step phases were analyzed. Research by A. Gawłowska et al. [8] similarly describes a person’s upper body movement using different sets of prostheses. To obtain kinematic data, a motion capture system OptiTrack Flex 13 (NaturalPoint, Corvallis, OR, USA) was used. Average movement speed and average vertical ground reaction force were also calculated. Present-day practice is more focused on angles benchmarking. It can be not only joint angles but, e.g., abduction and adduction or flexion and extension [9,10]. These studies illustrate motion capture system benefits and provide numerical results from experiments. However, an urgent task is to obtain a technique that helps to decrease the time of diseases diagnosis, as well as to adjust the applied therapy, depending on the current state of the patient [11,12].

Considering clusterization approaches, the proposed method allows taking into account the form of angle time series. Thus, the most common approach is to use peaks of angle functions [13] or even average values [14] for clusterization. There are more complicated methods to form data for clusterization [15], but that involves manual processing or questions the accuracy of automatic processing. The proposed parameters describe change range (in different part of gait phase) of some kinematic parameter (e.g., peaks). Such description is rather sketchy and does not take into account the curve form of kinematic parameter time-series. The proposed approach offers a solution. Obviously, the method or/and the order of approximation influence the quality of the following data processing, and this topic should also be investigated.

Therefore, in most articles, the researchers aim to analyze time-series of measured data and piecewise linear (usually constant) functions used for clusterization. The research is focused on convolution of the time-series data to parameters vector using higher order polynomial interpolation. Such technique allows obtaining the classification of patients by kinematic parameters, and the approach allows segregate patients in cluster groups for the following diagnostics.

## 2. Materials and Methods

### 2.1. Experiment Design

**Figure 1:**
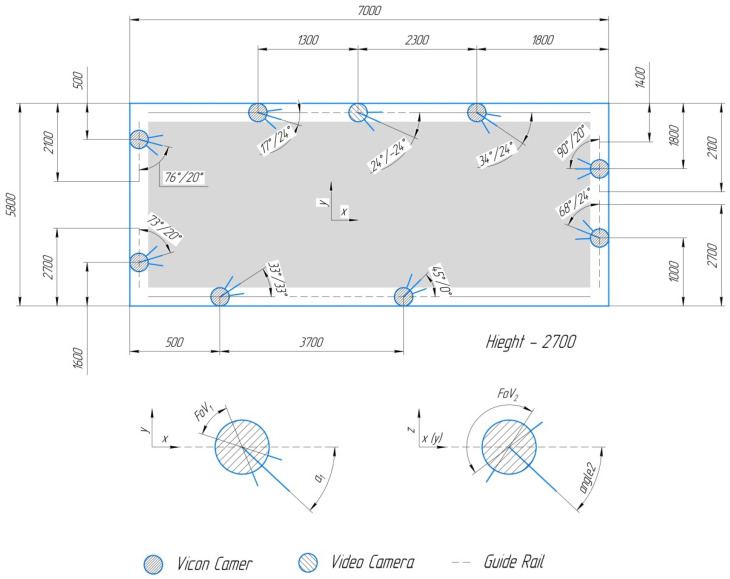
Arrangement of cameras on the working area.

Optoelectronic motion capture is widely used in biomechanics. Vicon is a system consisting of several Vicon Vero 2.2 digital infrared cameras (Vicon Motion Systems, Oxford, UK) with adjustable lenses and focus, and elaborated software for primary data processing and visualization Vicon Nexus 2.9.3. The scheme of cameras mounting is shown in Figure 1. Angle orientation is set by two angles a_1_ and a_2_ and shown in Figure 1. The field of view of Vicon Vero 2.2 digital infrared cameras was 50.1° × 98.1° (FoV_1_ and FoV_2_ in Figure 1 respectively). In Figure 1, the area covered by cameras is marked by the gray area.

Calibration and synchronization were carried out using the Active Wand calibration marker (Vicon Motion Systems, Oxford, UK). The standard video was obtained through a Sony camera. Kinematic data were collected at a sampling rate of 100 Hz and transferred to a personal computer using Vicon Nexus 2.9.3 software. The walkway was about 4 m.

**Figure 2:**
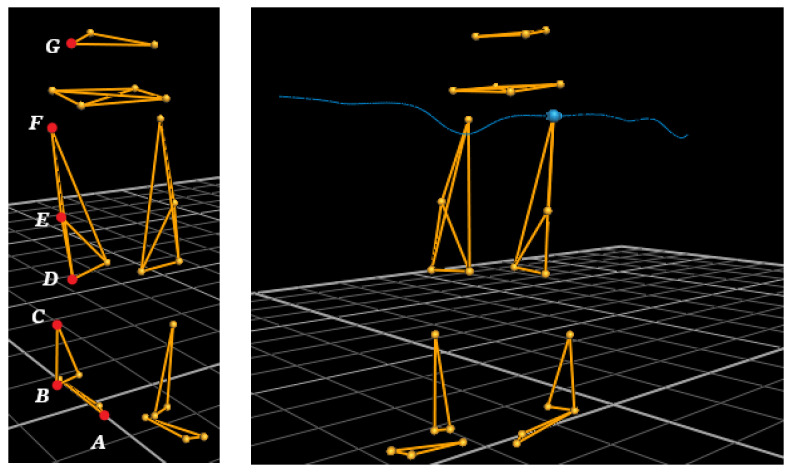
Visualization of the skeleton in the program Vicon Nexus 2.9.3.

The study involved six healthy subjects: 3 men and 3 women aged 20–26 years (mean age 24.16 years; SD 1.83), mean height 1.73 m (SD 0.07), and mean weight 69.5 kg (SD 14.6). The study protocol was approved by the Local Ethics Committee of the Federal State Autonomous Educational Institution of Higher Education KFU (protocol No. 12 dated 18 September 2018). All subjects were in socks and thin, tight clothing. A total of 27 reflective markers (14 mm diameter sphere) were placed on the following anatomical regions: calcaneus, toe, first metatarsal head, outer lateral malleolus, inner medial malleolus, tibia, outer or inner knee, femur, pelvic bones, and ilium. Markers’ location scheme is shown in Figure 2.

To increase the number of observations, the subjects performed different types of gait. The subjects walked straight. Each subject carried out several gait types: casual walking (T_1_), run (T_2_), and additionally imitation of a non-standard gait, including shuffling (T_3_), lameness (T_4_), clubfoot (T_5_), walking from the heel (T_6_), rolling from heel to toe (T_7_), walking with hands in pockets (T_8_), and catwalk (T_9_). Each type of gait was retested three times. The subjects did not have any vascular disease, skin problems, or balance limitations. They did not have operations on their lower limbs or serious injuries. The subjects were marked by letter F or M (depending on sex) and number. Non-standard gaits were used by subjects to increase the number of observations. Additionally, it allows to analyze the deviations in gait parameters in case of simulations.

The data from the cameras were transferred into Vicon Nexus 2.9.3 software. For the primary check, 3D reconstruction was used. It allows identifying and removal artifacts in the record. The following steps were taken: carry out identification for each marker on the screen; build a skeleton using the created markers; create areas (for example, foot, calf, thigh, etc.); create links between areas; calibrate this skeleton for each frame; and import a .c3d file. The created skeleton can be saved and successfully applied to the tracing. Thus, a three-dimensional array of x, y, z coordinates was obtained. It is possible to restore the trajectory of markers in case of their loss [16,17,18,19].

### 2.2. Data Processing

The input data are presented in the following structure:

(1) $$\left\{t_{i},m_{i1}\left(x_{i1},y_{i1},z_{i1}\right),\;\ldots ,m_{\mathrm{in}}\left(x_{\mathrm{in}},y_{\mathrm{in}},z_{\mathrm{in}}\right)\right\},i=\bar{\left(1,m\right),}$$

where t_i_—time data, m_i_—marker data, x_i_, y_i_, z_i_—marker coordinates, m—number of frames, and n—number of markers.

The X and Y axes were placed in the horizontal plane. The Z-axis was normal to the plane X-Y. The subject moved in the X-Z plane. The data were used to assess joint angle estimation [20].

The algorithm performing the following operations:

(1) Reading a file in .c3d format;
(2) Data visualization;
(3) Dividing the entire dataset into steps (touching the toe of the floor was step completing criteria);
(4) Assignment the stance phase and the swing phase at each step by searching for the local minimum of the heel movement trajectory [21].

The output of the aforementioned algorithm is an indices array, which allows dividing the data into steps and step phases. The developed software makes it possible to calculate the stride length, maximum and average height, and swing around the movement. These operations were calculated using a local minimum in projection onto each of the three orthogonal planes. Vectors constituting the required angles can be found by equations:

(2) $$\left\{\begin{array}{c} \bar{AB}=\bar{B}-\bar{A}\\ \bar{BC}=\bar{C}-\bar{B}\\ \bar{CD}=\bar{D}-\bar{C}\\ \bar{DE}=\bar{E}-\bar{D}\\ \bar{EF}=\bar{F}-\bar{E}\\ \bar{FG}=\bar{G}-\bar{F} \end{array}\right.$$

Next, the angles in the hip, knee, and ankle joint at each moment of time can be calculated according to the equations [6]:

(3) $$\left\{\begin{array}{c} ∠ABC=\arccos\left(\frac{\bar{AB}\cdot \bar{BC}}{\left|\bar{AB}\left|\cdot \right|\bar{BC}\right|}\right)\\ ∠CDE=\arccos\left(\frac{\bar{CD}\cdot \bar{DE}}{\left|\bar{CD}\left|\cdot \right|\bar{DE}\right|}\right)\\ ∠EFG=\arccos\left(\frac{\bar{EF}\cdot \bar{FG}}{\left|\bar{EF}\left|\cdot \right|\bar{FG}\right|}\right) \end{array}\right.$$

**Figure 3:**
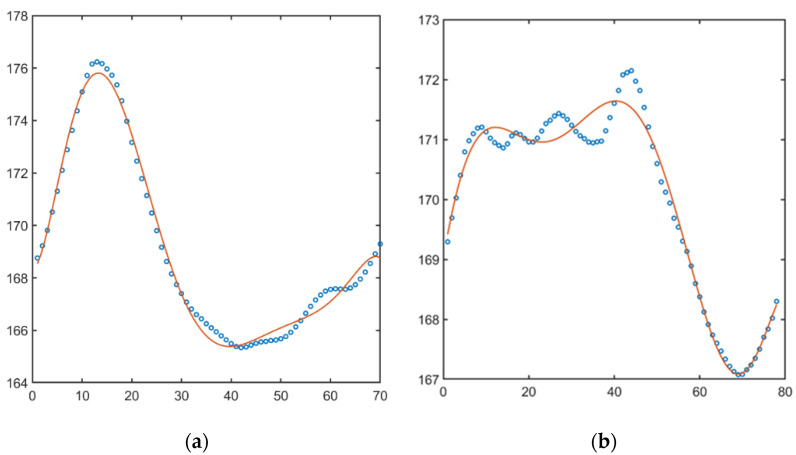
Change in the hip angle in swing phase (**a**), stance phase (**b**).

**Figure 4:**
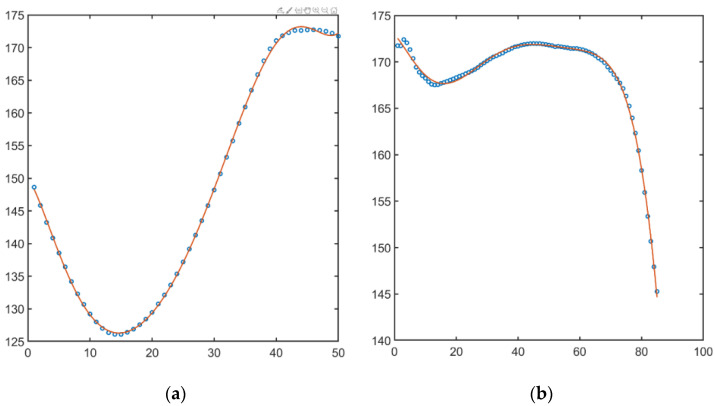
Change in the knee angle in swing phase (**a**), stance phase (**b**).

The obtained values of the angles (3), divided by phases, were saved in a file for further processing. To study the pattern of the movement, the changes in the angles in the stance phase and the swing phase were approximated by a seventh-degree polynomial function [22]. In Figure 3, initial data values (blue bubble) and polynomial approximation of swing and stance phases (orange lines) in knee angle are shown. Figure 4 illustrates the same but for hip angle. As a result of the operations, approximation coefficients were found for each record.

The next step was high-dimensional clustering for received data. As a result of the operations, approximation coefficients were found for each record. The main idea is to divide all dataset into groups with similar features. For clustering, the k-means method was used. The algorithm seeks to minimize the total square-law deviation of cluster points from the centers of these clusters.

(4) $$\overset{k}{\underset{i=1}{\sum }}\underset{x\in \mathrm{Si}}{\sum }\|x-\mu_{i}{\|}^{2}\to \min,$$

where k—number of clusters, S_i_—the full dataset, μ_i_—the cluster center.

The Euclidean distance was used in Equation (4). To determine the optimal number of clusters, the Calinski–Harabasz criterion was applied [23,24,25].

Angle results were averaged for all steps. To estimate deviation in movement, standard deviation was used. In this case, the resulting distribution can be presented as follows [6]:

(5) $${\;\bar{\varphi }}_{\pm }\left(\tau \right)=\mathrm{mean}\left(\varphi \left(\tau \right),N_{\mathrm{step}}\right)\pm \mathrm{std}\left(\varphi \left(\tau \right),N_{\mathrm{step}}\right),$$

where φ(τ)—angle function, mean (·, k) and std (·, k)—mean and standard deviation of function according to steps, N_step_—number of steps.

To quantify the volume of motion degree, the following equation was used to calculate the range of motion [6]:

(6) $${\;\bar{\varphi }}^{m}=\max\left({\;\bar{\varphi }}_{+}\left(\tau \right)\right)-\min\left({\;\bar{\varphi }}_{-}\left(\tau \right)\right)$$

Another parameter for estimating range of motions is the calculation of the triangle area. The vertices of triangle are the toe position at the initial moment of time; toe position at maximum height; and the hip position at the initial moment of time. The area of a triangle can be calculated as half of the vector product:

(7) $$S=\frac{1}{2}\|\left(\max({\;\vec{m}}_{i}^{X}\cdot {\;\vec{k}}_{i})-{\;\vec{m}}_{i_{j}^{\mathrm{start}}}^{X}\right)\times \left({\;\vec{m}}_{i_{j}^{\mathrm{start}}}^{X}-{\;\vec{m}}_{i_{j}^{\mathrm{start}}}^{Y}\right)\|$$

### 2.3. General Pipeline

For further analysis, the code was implemented in MATLAB software. Let us introduce the general pipeline for data processing (Algorithm 1).
**Algorithm 1. Data processing****Input:** motion capture data *Data* **Output:** parameter vectors *vParam* 1. Load *Data* in structure format according to (1) 2. *Data*(*step*) ← Divide *Data* by steps 3. **For** each *step* 4.   *Data*(*step, phase*) ← Divide *Data* by step phases 5. **End for** 6. **For** each *step* and *phase* 7.   *Angle*(*step, phase*) ← Calculate joint angles by Equations (2) and (3) 8.   *vParam*(*step, phase*) ← Approximate *Angle*(*step, phase*) 9.   *vParam*(*step, phase*) ← Calculate volume of motion by Equations (6) and (7) 10. **End for**

Then, the clusterization (4) was performed for parameter vectors *vParam* for all datasets.

## 3. Results and Discussion

**Figure 5:**
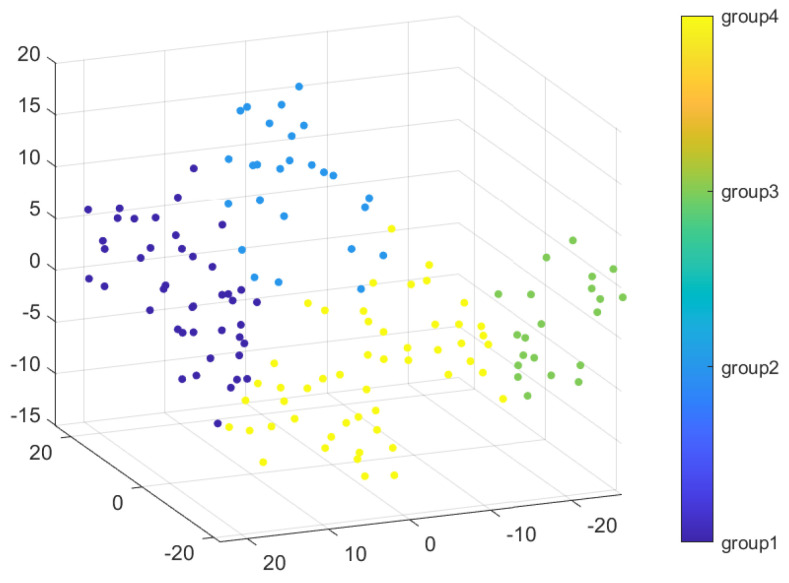
Cluster visualization for four groups.

For each record of each subject and each gait type, all aforementioned calculations were carried out (according to Algorithm 1). Then, clusterization by the coefficients of the approximating polynomials was carried out. As a result of clustering, the dataset was divided into 4 groups. Using t-Distributed Stochastic Neighbor Embedding, clusters are illustrated and shown in Figure 5.

Therefore, two subjects F_1_, F_2_, F_3_, and M_3_ had robust results via gait type. Subjects M_1_ and M_2_ appear in the 1st and the 4th cluster groups. For each cluster group, numbers of gait type records were counted. The gait types with maximum records amount for each cluster group are presented in Table 1. The 1st group consists of run (T_2_), walking from the heel (T_6_), and walking with hands in pockets (T_8_). Shuffling (T_3_) and walking with hands in pockets (T_8_) were in the 2nd group. Normal walk (T_1_) and catwalk (T_9_) were both in the 4th group. According to the gait type, the 3rd cluster group major consists of running type (T_2_).

Average hip angle in the swing phase for group 1 was equal 169° ± 2°, for group 2—161° ± 3°, for group 3—155° ± 5°, for group 4—165° ± 4°. Average value of the hip angle in the stance phase for group 1 was equal 168° ± 2°, for group 2—161° ± 2°, for group 3—161° ± 4°, and for group 4—167° ± 3°. Average knee angle in the swing phase for group 1 was equal 153° ± 4°, for group 2—150° ± 4°, for group 3—139° ± 8°, and for group 4—147° ± 5°. Average knee angle in the stance phase for group 1 was equal 163° ± 4°, for group 2—161° ± 5°, for group 3—153° ± 5°, for group 4—161° ± 4 ° (all data presented in Table 1).

**Figure 6:**
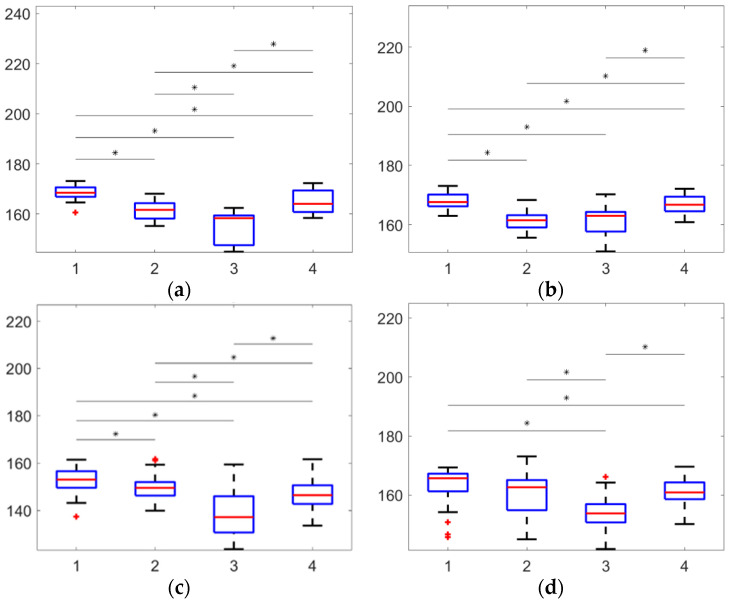
Data distribution in clusters: (**a**) hip angle value (°) in the swing phase, (**b**) hip angle value (°) in the stance phase, (**c**) knee angle value (°) in the swing phase, (**d**) knee angle value (°) in the stance phase; significantly difference pairs are marked by *, outliers marked by +.

Statistical differences between all groups in the case of the swing phase (*p* < 0.05) were shown by paired-samples *t*-test. For stance phase, partial statistical differences (*p* < 0.05) were found. In Figure 6, box plot and pair statistical differences are shown for hip angle in the swing phase (Figure 6a), hip angle in the stance phase (Figure 6b), knee angle in the swing phase (Figure 6c), andknee angle in the stance phase (Figure 6d).

It should be noted that the average angles in the swing phase change in proportion to the average angles in the stance phase for the hip and knee angles. This information, together with the results of the paired-samples *t*-test, makes it possible to conclude about the veracity of the distribution into groups. The stance phase takes about 60% of the stride cycle, which helps to align the body. Perhaps, for this reason, there is no statistical difference between some groups in the stance phase.

**Figure 7:**
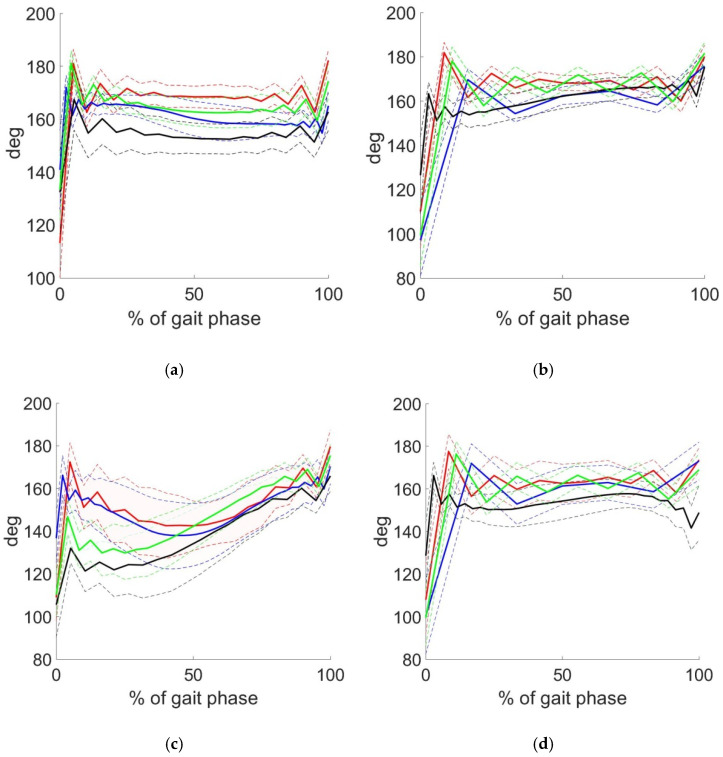
Changes in the hip and knee angles (°) in phases for clusters: (**a**) hip angle in the swing phase, (**b**) hip angle in the stance phase, (**c**) knee angle in the swing phase, (**d**) knee angle in the stance phase. Color designations: red—1 group, blue—2 group, black—3 group, green—4 group.

In Figure 7, hip and knee angle results are presented. Here, the thick line—mean values of angle, dashed line area—standard deviation (function φ ± (τ) in Equation (5)) of an angle [23,24,25]. The change in the hip angle in the swing phase (Figure 7a), and in stance phase (Figure 7b), the change in the knee angle in the swing phase (Figure 7c) and in stance phase (Figure 7d) are shown. Curves have different colors: group 1 is highlighted in red; group 2—blue; group 3—black; group 4—green. All curves in each of the four images are located to a greater degree in the same order.

**Figure 8:**
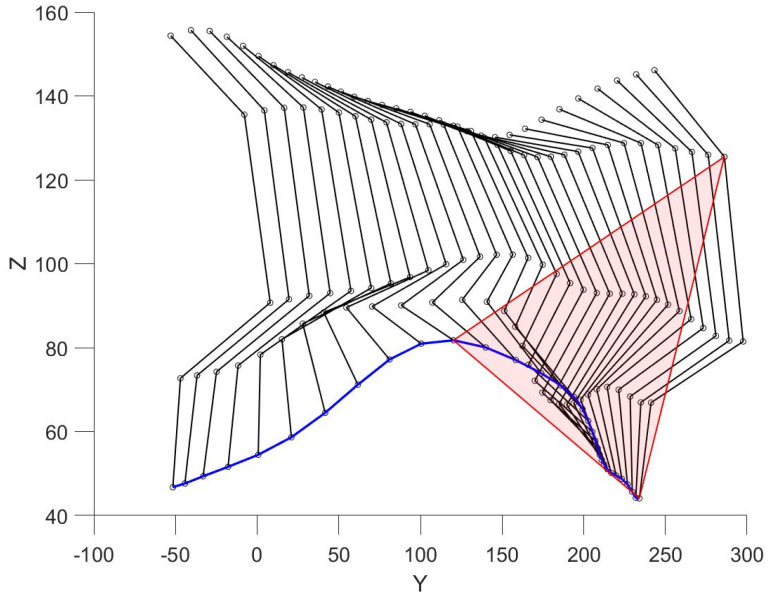
Constructing a triangle.

Figure 8 shows the position of the right leg markers for one step in each frame. The blue line is the trajectory of the ankle movement. Red lines enclose a triangle describing the subject’s range of motion (7). This method of assessing movement can be used as an additional criterion for the distribution of subjects into clusters.

After applying the approach described above, clustered groups of gaits were found. Therefore, classification can be used to allocate new data of subjects to existing groups. This will speed up the process of identifying health deviations of new test subjects in future.

Currently, there are new techniques for motion capture and analysis of the kinematics of the movement of an object [26,27,28]. Each researcher strives to make his concept more optimal, easier to use, and cheaper. Therefore, the use of 2D technologies such as video cameras [29,30] is being actively introduced. On one hand, it is easier and cheaper, but still there is no protocol for such measurements [28]. That leads to different number of data deviation and questions the received results. Of course, general information such as body position and orientation can be found by 2D technologies. In contrast, 3D analysis equipment such as Vicon transmits the most accurate marker position. Placing the markers on anatomical positions allows to calculate joint angles more accurately. Moreover, while using video, it is possible to calculate only a plane angle, in contrast to using Vicon. When it comes to tracking the dynamics of a patient’s condition and adjusting his therapy, it is necessary to obtain results of high accuracy.

Other studies [31,32] have shown the possibility of preventing a patient from falling through real-time gait analysis. The proposed method was evaluated by recording the normal gait and simulating the pathological gait of the subjects. To determine the phases of the step, it was proposed to use pressure sensors installed in the shoe linings. This solution allows to simultaneously determine all phases of the gait and the distribution of the load on the feet, which is important when assessing gait discoordination. This technique provides a minimum level of discomfort for the object, since there are no direct contacts between the sensor surface and the human body in the system. The patient can use this measuring equipment all the time, which means continuous monitoring is possible.

Conducting research in the field of kinematic analysis of motion is possible using various devices and techniques. Each of them has its advantages and disadvantages; however, some of them are more applicable in a particular clinical case. Combination of video fixation and feet pressure sensor is optimal for full-scale research, but it limits by laboratory capabilities. Researchers should emphasize data processing taking into account not only the physics of measurement equipment but mathematical formalism.

Comparing to other studies, it can be noticed that there is dispersion in patterns. It can be explained by common limitations. Not only clusterization methods [14] but input variables greatly influence the results. Usually easily measured parameters (velocity, cadence, stride length, etc.) are used [13]. Of course, directly increasing the number of parameters can solve the problem. On one hand, dependent variables can appear; on the other hand, it can reduce robustness. Such problems can be solved by dimensionality reduction methods [14], but still there is no general solution. As for clinic practice, there are also obstacles. Direct correlation between clusters groups (received by gait data) and injuries or diseases does not always appear [15,33]. Studies offer ways to meet the challenges: increase data, increase number of gait phases, etc. Nonetheless, gait parameters for clustering are a priori selected, and usually describe time series in terms of constants peaks, excursions, ranges, etc.) [34,35].

Regarding the proposed approach, the time series form was used for clusterization. We cannot be sure of the stability of the received groups and do not pretend that they will be the same in case of data augmentation. The research was focused on method and software development. Conducted research shows results which can be used in the future. However, more importantly, the paper brought up a point of methods for processing time-series for clusterization. Focused on interpolation results, a case with minimal deviation from interpolation (see Figure 4a,b) and a case with deviations (see Figure 3b) were found. We can only question, how does it influence the final clusterization results? Herewith, two obvious solutions can be pointed out: applying more complicated functions for interpolation (e.g., non-uniform rational B-spline) or using methods of topological data analysis.

## 4. Conclusions

This article provides a detailed description of the methodology for distributing data into groups with different characteristics. It can be helpful to speed up diagnosis of the patient’s disease and start the treatment in the shortest possible time. Undoubtedly, this technique can be applied to all patients with musculoskeletal system problems. Moreover, the results obtained indicate the possibility of using the technique for prosthetics to monitor the progress of rehabilitation and adjust the treatment method. An approach for data clusterization was presented. For this purpose, angle data were interpolated and the interpolation coefficients were used for clustering the data. The data were processed and four cluster groups were found. Typical angulograms for cluster groups were presented.

The proposed method has obvious limitations involving the degree of approximation. The noisy input data can influence polynomial vector and consequently clustering results. To improve the robustness of the method, it is planned to try topological data analysis. Despite the limitations, the proposed method allows to get objective results.

## Figures and Tables

**Table 1:** Cluster groups results.

Group	Hip Angle		Knee Angle		Subject ID	Gait Type ID
	Swing, °	Stance, °	Swing, °	Stance, °		
1	169 ± 2	168 ± 2	153 ± 4	163 ± 4	F_1_, M_1_, M_2_	T_2_, T_6_, T_8_
2	161 ± 3	161 ± 2	150 ± 4	161 ± 5	F_2_	T_3_, T_8_
3	155 ± 5	161 ± 4	139 ± 8	153 ± 5	F_3_	T_2_
4	165 ± 4	167 ± 3	147 ± 5	161 ± 4	M_1_, M_2_, M_3_	T_1_, T_9_
